# Non-Neutralizing Antibodies Directed against HIV and Their Functions

**DOI:** 10.3389/fimmu.2017.01590

**Published:** 2017-11-20

**Authors:** Luzia M. Mayr, Bin Su, Christiane Moog

**Affiliations:** ^1^INSERM U1109, Fédération de Médecine Translationnelle de Strasbourg (FMTS), Université de Strasbourg, Strasbourg, France; ^2^Beijing Key Laboratory for HIV/AIDS Research, Center for Infectious Diseases, Beijing You’an Hospital, Capital Medical University, Beijing, China

**Keywords:** HIV-1 infection, non-neutralizing antibodies, antibody functions, antibody-dependent cellular cytotoxicity, Fc-receptor-mediated inhibition

## Abstract

B cells produce a plethora of anti-HIV antibodies (Abs) but only few of them exhibit neutralizing activity. This was long considered a profound limitation for the enforcement of humoral immune responses against HIV-1 infection, especially since these neutralizing Abs (nAbs) are extremely difficult to induce. However, increasing evidence shows that additional non-neutralizing Abs play a significant role in decreasing the viral load, leading to partial and sometimes even total protection. Mechanisms suspected to participate in protection are numerous. They involve the Fc domain of Abs as well as their Fab part, and consequently the induced Ab isotype will be determinant for their functions, as well as the quantity and quality of the Fc-receptors (FcRs) expressed on immune cells. Fc-mediated inhibitory functions, such as Ab-dependent cellular cytotoxicity, antibody-dependent cellular phagocytosis, aggregation, and even immune activation have been proposed. However, as for nAbs, the non-neutralizing activities are limited to a subset of anti-HIV Abs. An improved in-depth characterization of the Abs displaying these functional responses is required for the development of new vaccination strategies, which aim to selectively trigger the B cells able to induce the right functional Ab combinations both at the right place and at the right time. This review summarizes our current knowledge on non-neutralizing functional inhibitory Abs and discusses the potential benefit of inducing them *via* vaccination. We also provide new insight into the roles of the FcγR-mediated Ab therapeutics in clinical trials for HIV diseases.

## Introduction

A strong antibody (Ab) response is mounted following HIV infection but most Abs targeting the HIV have little neutralizing capacity. Upon humoral immune activation *via* infection, B cells undergo somatic hypermutations and isotype switching of the immunoglobulin gene in order to enhance the efficacy of the Ab response against the specific antigen (1). B cells can then differentiate into long-lived plasma cells (2). However, most of the B cells induced are directed against decoyed immune-dominant epitopes that have no or low antiviral function. The targeted epitopes are either useless for antiviral activity (directed against unfolded glycoprotein that are not present on infectious viruses) or against epitopes able to efficiently and quickly mutate to escape from the immune response. Only 10–20% of infected individuals are able to mount a B-cell response leading to the production of broadly neutralizing Abs (bnAbs). These bnAbs represent, therefore, only a minor amount of the humoral Ab response induced following HIV infection. They have specific characteristics: they are produced from B cells that undergo unusually long maturation steps with extraordinary levels of somatic mutations compared to germline and display long heavy chain complementarity-determining regions 3 to be able to bind masked epitopes. This allows the development of Abs that target specific antigens with high affinity (2).

In addition to germline mutation, the consecutive immunoglobulin class switching will change the Ab isotype (3). This Ab isotype switch is also determinant for its gain of function. The heavy chain constant region determining the Ab isotype will not only impact the neutralization capacity (*via* the Fab domain) but also play a crucial role on the Ab effector functions (*via* the Fc domain). In fact, the heavy chains define the Fc domain that will directly modulate the Fc-mediated inhibitory functions. These functions will greatly influence the further immune response. Interestingly, Fc-mediated inhibitory function was detected not only on neutralizing Abs (nAbs) but also on some specific Abs lacking neutralizing activity, therefore, called non-neutralizing inhibitory Abs (4) [reviewed in Ref. (5–11)].

*In vivo*, the Fc-mediated functions are now being addressed. It is well documented that the Fc-mediated effector functions contribute to Ab-mediated protection against HIV-1 for bnAbs (5–8, 12, 13). Two recent studies have tracked virus replication after early experimental mucosal infection and passive protective bnAb therapy (14, 15). Liu et al. showed that, in animals pre-treated with bnAb PGT121 1 day before challenge with high-dose mucosal SHIV, early viral foci are detected at the distal site of infection before complete virus clearance (14). These results showed that bnAbs are able to eliminate the infected cells if some virus escapes from the neutralization of infection. Moreover, Hessell et al. found that early short-term post-exposure treatment with a cocktail of bnAbs VRC07-523 and PGT121 in newborn macaques, 1 day after oral SHIV_SF162P3_ challenge can intercept replicating viral foci established by day 1 (15). This study demonstrates that passive immunotherapy by Ab can eliminate viral foci and thereby prevent the establishment of viral reservoirs (14, 15). These two studies exploring early virus replication in the presence of nAbs clearly demonstrate that HIV escaping from neutralization can infect cells at a distal site of virus inoculation and be subsequently eliminated by bnAbs. They reveal that the complete lack of infection is not mandatory to obtain protection by nAbs. The discovery that nAbs can eliminate a few foci of infected cells is extremely useful for the vaccine field as this type of activity cannot be referred to as neutralization. The mechanism by which Abs ensure clearance of infected cells is not known but obviously these additional observations reinforce the potential role of Fc-mediated functions in the protective mechanisms of bnAbs. These results open a complete new area of research for the development of protective Ab responses. Additional experiments are now required to define the mechanism of infected cell clearance. In particular, increased analysis of the Ab protection at very early time points following challenge will help to identify the multiple inhibitory functions displayed by bnAbs.

The role of Fc-mediated functions of Abs lacking the broadly neutralizing capacity in HIV protection is still a matter of debate. Importantly, specific Fc-mediated functions of non-neutralizing Abs (non-nAbs) are the only correlates of protection against infection observed in the RV144 vaccine trial conducted in Thailand (16–19). Still, how non-nAbs have contributed to protection remains unclear. In the non-human macaque model, the non-nAbs have shown some trends of decreased viral load or decreased number of transmitted founder viruses (20, 21). The exact mechanisms leading to this lower infection rate is not known, but again indicated that non-nAbs may participate in protection. Active immunization with HIV-1 vaccine candidates suggests that weakly neutralizing or non-nAbs protect by using Fc-mediated effector functions, albeit with a much lower dynamic range as for passive immunization with bnAbs (22). New tools, such as knockout mice or Abs engineered to abrogate or enhance certain functions, were recently developed. These technologies recently paved way for the demonstration of the role of Fc-mediated functions (23). Treatment with a non-nAb directed against the principal immunodominant domain of gp41 allowed for the selection of a recurring HIV mutation within the CD4 binding site in a totally Fc-dependent manner (23). These data are consistent with the hypothesis that a high titer polyclonal anti-envelope (env) non-nAb response may be sufficient to reach low levels of protection against HIV. Future directions need to more precisely characterize the functions and Ab characteristics needed to achieve such protection.

The identification of these additional non-neutralizing inhibitory Abs opens a whole new area of research. Functions involving the Fc domain of Abs can occur simultaneously, sequentially, and can sometimes be conflicting with other Ab functions. They were shown to contribute to the overall protective effect of Abs and to an efficient humoral immune response (5, 8–10, 12, 13, 20, 21, 23–25). This review will discuss the opportunity, difficulties, limitations, and parameters influencing these Fc-mediated Ab functions.

## Functional Activities of Abs Capturing Infectious HIV Particles

HIV-specific Abs are directed against numerous epitopes of the HIV glycoprotein, but only few are accessible as a quaternary structure of the functional trimeric envelope. Among them, five hotspot epitopes were shown to be involved in HIV neutralization (26, 27). Even so, Abs to additional epitopes were shown to bind to infectious viruses either by targeting additional epitopes on the trimeric env or non-functional env spikes expressed on HIV particles. These additional Abs, although not neutralizing, are able to bind and capture infectious virus, form immune complexes and/or virus/Ab aggregates, therefore leading to additional inhibitory functions.

## HIV Inhibition by Aggregation

Formation of virus aggregates is a very basic mechanism of inhibition leading to the decrease of virus infectivity (28–32). The aggregates are formed by a network of Ab/virus interactions, where the virus is trapped. This leads to virus inactivation by limiting the distribution and accessibility of available pathogens, decreasing their motility or disrupting their integrity. This mechanism applies to Abs binding to numerous epitopes exposed at the surface of the virus particle. Aggregation more likely occurs with polymeric IgA that are able to dimerize *via* their Fc domain and IgM displaying pentameric forms. Indeed, inhibition by aggregation was proposed for the exceptional protective effect observed with IgA1 (33). In this study, a correlation was observed between the binding capacity of the anti-HIV IgA1 subclass Abs and the protective effect on rectal experimental challenge (33). For IgG, aggregation occurs by the recognition of two distinct epitopes/virions entities. This activity, therefore, usually has a dome-shaped relationship to the Ab concentration, declining at higher occupancies when it becomes improbable that a free paratope of an Ab molecule already bound to one virion can find a free epitope on a second virion. In the female reproductive tract containing cervical mucus, HIV aggregates will be trapped more efficiently as free virus particles (34). Moreover, the immune complexes formed may be retained efficiently in the mucus by their binding to MUC16 *via* the Fc domain of IgG Abs (24). In addition to this mechanic inhibition of HIV by aggregate formation, more complex mechanisms involving a further binding of the Abs to the Fc-receptor (FcR) expressed on the surface may take place.

## The Role of FcRs

Fc-mediated inhibitory activity is entirely dependent on the capacity of Abs to trigger FcRs. These FcRs have to interact with the Fc domain of the Abs to trigger the Fc-mediated functions. Based on their homology, three classes of FcγRs have been described (FcγRI, II, and III). The distinct family members, including FcγRI, FcγRIIa, FcγRIIb, FcγRIIIa, and FcγRIIIb, are differentially expressed on the surface of immune cells, such as B cells, dendritic cells (DCs), NK cells, macrophages, neutrophils, eosinophils, and basophiles (35–39). They differ in their Ab affinities, favoring certain IgG subtypes depending on their amino acid sequences. This differential binding capacity, depending on the Ab isotype and the FcR genotype and its expression on the cell modulates the Ab activities and their capacity to activate or inhibit FcR-expressing cells. Therefore, the different FcR polymorphisms of the host need to be taken into consideration when analyzing FcR-mediated functions of Abs.

Single-nucleotide polymorphisms (SNPs) have been described to occur in FcγRIIa, FcγRIIIa, and FcγRIIb at protein positions 131, 158, and 232, respectively, while human FcγRI was not found to be polymorphic. Since these SNPs affect FcR expression and IgG isotype binding leading to distinct effector functions, they can influence HIV vaccine efficacy, infection risk, and disease progression. For example, specific polymorphisms at the FcγRIIa (change from H to R at position 131) and the FcγRIIIa (change from V to F at position 158) gene loci have been associated with an HIV vaccine benefit (40). Li et al. described that subjects carrying a SNP in FcγRIIc (126C>T) were associated with a significant prevention of infection with an AE HIV-1 strain in the RV144 vaccine clinical trial (41). On the contrary, a small study that compared the FcγRIIa and FcγRIIIa genotype profiles of 73 patients that were able to control HIV with progressor patients did not find any difference in genotype frequency (42). The role of the different FcR polymorphisms and how it will impact on the overall HIV immune response is not known. Therefore, future research will need to assess in more details the role of FcR polymorphisms of the host on HIV infection and HIV vaccine development.

## Antibody-Dependent Cellular Phagocytosis (ADCP)

Antibody-dependent cellular phagocytosis, which relies on phagocytes to internalize and degrade Ab-opsonized pathogens, is a well-described immune process. Abs coated to pathogens *via* their Fab domain will bind with their Fc domain to the FcR expressed on monocytes, macrophages, and neutrophils to increase rapid elimination of the microorganisms. In the case of HIV, phagocytosis of immune complexes *via* the Fc domain of the nAbs was found to be associated with protective activity in the macaque model (43–45) and, recently, phagocytosis by macrophages or activated neutrophils was proposed to play a significant role in human tissues, even though it is yet unknown how exactly this inhibition occurs (46). Interestingly, this activity was also described for non-nAbs able to form immune complexes. It was shown that for some HIV-specific Abs, the binding *via* the Fab domain, on the one hand, and the binding to an antigen-presenting cell (APC) *via* the Fc domain, on the other hand, leads to efficient inhibition of HIV replication of the APCs (4). Phagocytosis by cell lines was shown using different HIV-specific Abs and gp120-coated beads (47) and when these cell lines were engineered to express different FcRs, the FcR-mediated inhibitory function of Abs was partially recovered. This type of activity relies on multiple Abs, able to form immune complexes and especially for Abs directed to the HIV gp41 epitope (33). Although HIV inhibition by phagocytosis of the immune complex could not be demonstrated using this FcR-expressing cell line, it was proposed that immune complex binding of FcγRI provides a kinetic advantage for gp41 nAbs against partially cryptic epitopes (33). An alternative mechanism may be proposed based on the observation that virus co-localizes with Abs and FcRs at the surface of APCs for a prolonged period. In this case, HIV captured at the cell surface *via* FcRs is deviated from the infection process, which requires binding to receptor/co-receptor for fusion with the cell membrane.

## Immunological Ab Function

Antigen-presenting cells are specialized cells devoted to phagocyte immune complexes *via* their FcRs. This phagocytic process is much more efficient than the direct phagocytosis of pathogen by endocytosis. This mechanism of Fc-mediated phagocytosis of immune complexes will lead to an optimized induction of the adaptive immune response by APCs. In this regard, Abs forming the immune complexes may directly participate in the induction of the adaptive immune responses required for prolonged protection. The contribution of Abs in the development of an adaptive immune response was first described in the cancer field (48). Abs targeting tumor antigens were shown to interact with immune cells through Fc-dependent mechanisms to induce adaptive immune responses (49–51).

Increasing body of evidence suggests that this mechanism may also apply following HIV infection. Noteworthy, *in vitro*, the presence of HIV/Ab immune complexes induces the maturation of human DCs, supporting immune activation (52–54). The stimulation of the adaptive immune response was also observed following nAb therapy in infected macaques (45, 55). An increase of specific B-cell responses following passive nAb transfer in a non-human primate (NHP) model was described by Haigwoog’s team (56). The immune complexes were able to activate T-cell immunity (57). More recently, human clinical data described the elicitation of host humoral responses in viremic subjects after a single injection of the potent anti-HIV nAb 3BNC117 (58). 3BNC117 immunotherapy was found to accelerate the level of neutralization breadth. Overall, these studies attribute an “immunogenic” role to Abs in that they may be able to induce primary and memory responses more efficiently than free viral particles or infected cells. Accordingly, Abs without neutralizing potency but able to form immune complexes may also lead to immune activation. Further investigations will be necessary to characterize the Abs involved in the implementation of an adaptive antiviral response, paving the way to new fields of applications.

## FcR-Mediated Inhibition of Cell-to-Cell HIV-1 Transmission

Noteworthy, APCs have been described as “Trojan horses” that, in addition to their capacity to mount an efficient immune response, will also facilitate the spread of HIV by efficient HIV transmission and dissemination to the surrounding CD4 T lymphocytes. Indeed, spread of HIV-1 infection through direct cell-to-cell HIV-1 transmission has been shown to be 100- to 1,000-fold more efficient than infection by cell-free virus, making a large and efficient contribution to HIV propagation and dissemination through the body (10, 59–61). Therefore, preventing cell-to-cell transmission of HIV-1 by specific Abs is crucial for inhibiting HIV-1 propagation. However, most *in vitro* neutralization assays and *in vivo* nAb protection experiments have been performed by using cell-free virus.

Studies analyzing the inhibition of cell-to-cell HIV-1 transmission by nAbs used diverse models of HIV-1 transmission, with different donor and target cells, various viral strains, and Ab and different readout for cell-to-cell transmission. Consequently, the results are divergent and controversial, some studied showing decreased Ab potential when HIV is directly transmitted to a target cell compared to inhibition of cell-free virus (62–72), whereas other studies showing similar inhibitory potential for cell-free versus cell-to-cell transmission (52–54, 73). Noteworthy, in comparative studies where the experimental design is normalized for the same replication capacity between cell-free or cell-associated virus and where the same primary target cells were used, identical Ab inhibitory activities were observed (52–54, 74). Under these conditions, cell-to-cell HIV-1 transmission from DCs/macrophages to CD4 T cells was inhibited to a similar extent as cell-free virus particles. Interestingly, similar results were described for antiviral compounds after normalization for virus replication and target cells (73, 75). These findings highlight the potential role of bnAb in protection from early HIV-1 transmission and rapid dissemination at mucosal frontlines if locally present early after sexual transmission.

As HIV-1 Abs can bind FcRs, Abs may inhibit HIV-1 transmission *via* FcR-mediated inhibitory activity. It was shown that non-neutralizing inhibitory Abs such as 246-D do not directly affect HIV-1 transmission from infected DCs to autologous CD4 T cells (54). Therefore, non-neutralizing inhibitory Abs were proposed to have no direct effect on HIV transmission. However, such Abs were shown to significantly reduce the percentage of infected DCs in DC-T cell co-cultures (54). For these non-neutralizing inhibitory Abs, a strong association was found between the FcγR-specific binding capacity, the inhibition of HIV-1 replication and the DC maturation. This suggests that the binding of these Abs to DCs triggers the maturation of these cells, resulting in lower levels of R5 virus replication (10, 54). Moreover, IgG-opsonized HIV-1 has been showed to impair provirus formation, p24 production and to decrease the long-term transmission rate to autologous non-stimulated CD4 T cells (76). These unconventional mechanisms of HIV inhibition detected in DCs but not in CD4 T lymphocytes may explain the lower levels of infection in the co-culture in the presence of non-nAbs. Therefore, these Fc-mediated inhibitory activities of Abs in DCs may participate in the overall diminution of HIV replication in DC–T cell HIV-1 transmission.

Altogether, the multiple Ab inhibitory activities should be taken into consideration for the study of the inhibition of cell-to-cell HIV-1 transmission. A better understanding of this FcR-mediated inhibition of HIV transmission is needed for future Ab-based therapeutics and protection strategies.

## Antibody-Dependent Cellular Cytotoxicity (ADCC)

Antibody-dependent cellular cytotoxicity, a complex and potent Fc-mediated effector function, requires the linking of an HIV-infected target cell to an immune effector cell *via* HIV-specific Abs. In this regard, Abs have to bind to HIV env, which is expressed on the surface of infected cells, *via* their Fab part and use their Fc domain to interact with FcRs expressed on the surface of effector cells, such as NK cells. This double interaction triggers the release of cytotoxic granules containing perforin and granzymes from the effector cells, leading to the death of the Ab-bound infected target cells.

Antibody-dependent cellular cytotoxicity as well as non-neutralizing anti-V1/V2 Ab induction was shown to correlate with reduced HIV-1 infection risk in the human vaccine trial RV144 and in several NHPs studies (16, 18, 77–79). The data strongly suggest for ADCC to be a significant mechanism of protection against HIV-1 *in vivo* (7, 10, 22, 80). Interestingly, non-neutralizing anti-V2 monoclonal Abs elicited in HIV-1-infected patients recently showed strong cross-reactive ADCC activity using different primary subtype B and C isolates as well as subtype B Transmitted/Founder viruses *in vitro* (81). This study reinforces the potential role of V2-specific Abs. However, as ADCC is a complex and multilayered activity, questions remain about which ADCC assay best reflects the biology of protection and shows the best correlation with *in vivo* studies. *In vitro* assays are difficult to carry out and the variability obtained between different ADCC assays developed in the HIV field is alarmingly high, due to different assay formats and readouts circulating in the field.

As the HIV env is conformationally highly dynamic and as different epitopes are exposed during the different phases of infection because of structural rearrangements, the window of opportunity for Abs to bind to their specific epitope in order to mediate ADCC might only be a few hours, during the viral entry and budding phases (7, 8, 82). Furthermore, different env forms (such as intact env, non-trimeric env, gp41 stumps, env peptides presented by the MHC, and so on) are expressed on infected cells depending on the Nef and Vpu accessory proteins present in the chosen virus type (primary virus, pseudovirus, infectious molecular clone). Also, HIV was shown to prevent the accumulation of env at the surface of target cells *via* a Vpu-mediated BST-2 antagonism (83). Noteworthy, the epitopes tackled by Abs with potential ADCC functions may differ from that involved in neutralization, opening the possibility of additional, enlarged, and distinct pattern of functional Abs. As a result, depending on the different env conformations, the recognition of specific epitopes will be influenced and have an impact on the ADCC results (84).

As ADCC relies on the capacity of the Ab to target infected cells, it could be proposed that by extension, Abs directed to all type of markers specifically expressed on infected cells may make the job. Therefore, targeting infected cells with Abs directed to FcRIIa, a marker recently identified on HIV cells reservoirs (85) or to specific markers of cell stress induced following infection (as NKG2D or MHC-E) may also participate in infected cell clearance. Another factor influencing ADCC outcomes and, thus, HIV disease progression are the target cells that carry out the lysis, which are predominantly NK cells. Their maturation and activation status as well as their subset distribution can vary widely in different tissues and according to the individual. Therefore, the activation of the ADCC target cells may be envisaged to enhance ADCC efficiency. Also, different polymorphisms on FcγRIIIa, expressed on NK cells, can impact their activation and ADCC activity (86).

## Antibody-Mediated Complement Activation

The complement system is an integral part of the innate immune system which has multiple effects, including opsonization, recruitment of inflammatory cells, and cell lysis/virolysis. Complement activation can occur through three distinct pathways: classical, alternative, and lectin, and is vital for both innate and adaptive immune responses (87–91). Complement activation results in the generation of C3 and C5 convertase complexes, which cleave C3 and C5, respectively, to generate the anaphylatoxin components C3a and C5a as well as the opsonin C3b, membrane attack complexes initiator C5b and, finally, to perforate the viral surface causing disruption and, thus, complement-mediated lysis (87, 88, 91).

Antibody-mediated complement activation by HIV has been widely studied over the years. The initially published studies on complement and HIV were conflicting (92, 93). Some reports said that the virus did not bind human serum complement unless Ab to the virus was present. Others suggested that the virus activated and bound complement spontaneously, even in the absence of Ab. The current knowledge, however, concludes that HIV has developed a sophisticated defense that protects the virus by failing to bind complement proteins. Indeed, virions bind complement poorly (especially the gp120 that is refractory to complement binding) (94). Moreover, HIV incorporates the human cell membrane complement down-regulatory molecules CD46, CD55, and CD59 during budding, thereby inhibiting complement-mediated damage to the virus. For this reason, the use of primary isolates produced by primary cells is absolutely mandatory for the study of complement-mediated effects. HIV also captures serum factor H to downregulate complement binding (95–97). On the other hand, HIV has evolved several mechanisms to exploit the complement system to facilitate the binding of HIV to target cells *via* CR2 or CD21 proteins, therefore leading to the enhancement of viral infectivity and the formation of virus reservoirs at different stages (98–104). For example, complement-mediated enhancement of HIV-1 by autologous non-nAbs obtained during acute HIV-1 infection was recently illustrated in *in vitro* studies (95, 98).

Interestingly, the role of complement activity of the Fc domain of nAb b12 evaluated in the non-human macaque challenge model revealed that a b12 Fc mutant defective for C1q binding and complement activation exhibited comparable activity to that of wild-type b12 (13). This indicates that complement is not required for optimal *in vivo* Ab protection against SHIV infection (13). Nonetheless, complement activation by V1V2-specific Abs was stronger and detected more frequently in RV144 with a reduced risk of HIV-1 infection than in two related trials, VAX003 and VAX004, for which no significant protection was observed (105). These results suggest that a certain level of Ab-dependent complement activity may have contributed in part to a modest protection against the acquisition of HIV-1 infection in the phase III RV144 HIV-1 vaccine trial. Together, complement can mediate a variety of biological functions, the relative contribution of virus lysis and enhancement in the tissue and in the periphery may differ and needs to be further investigated. Additional studies will be needed to define the role of complement activation and regulation in HIV infection and to unravel whether the beneficial or the detrimental effects of complement and Ab dominate *in vivo*. A possible balance of Ab-mediated immune responses, including complement activation, may be the key for the induction of *in vivo* protection against HIV.

## Conclusion

The plethora of additional Ab functions listed below demonstrates the extremely large potential of functional Abs. Therefore, there is no single mechanism or assay that has come to the front to predict vaccine efficacy. This is a major issue confronting researchers in the HIV field and it is also important for other cases of Ab-mediated protection against infectious diseases.

The Abs will be produced by B cell following an interplay of somatic hypermutations and isotype switching. The successive modifications leading to the maturation of the immune response is still poorly understand. Recently, the frequency of HIV-env-specific memory B cells correlated positively with the neutralization breadth in HLA-B*57+ HIV elite controllers but not in HLA-B*57-elite controllers (ECs), suggesting a very specific induction or preservation of HIV-specific memory B cells in these patients (106). However, the factors allowing the establishment of this efficient humoral response is not known.

The long-lasting persistence of HIV following infection demonstrated that the sole repetitive contact with an antigen is not sufficient to mount a humoral response able to generate functional Abs. What are the additional component necessary to induce the rearrangement necessary to obtain B cells producing Abs with the Fab domain that recognize the right epitope and the Fc domain with the best functionality? Even more enigmatic, which immunization protocol can trigger such a response? The in-depth characterization of the different Ab functionality is the first step toward the understanding on how to trigger such an efficient B-cell response.

## Author Contributions

LM, BS, and CM wrote the manuscript. CM revised the manuscript.

## Conflict of Interest Statement

The authors declare that the research was conducted in the absence of any commercial or financial relationships that could be construed as a potential conflict of interest.
